# Supplementary figures and images for: MOSAIK: A Hash-Based Algorithm for Accurate Next-Generation Sequencing Short-Read Mapping
Source: PLoS One. 2014 Mar 5;9(3):e90581. doi: 10.1371/journal.pone.0090581 (PMC3944147; doi:10.1371/journal.pone.0090581)

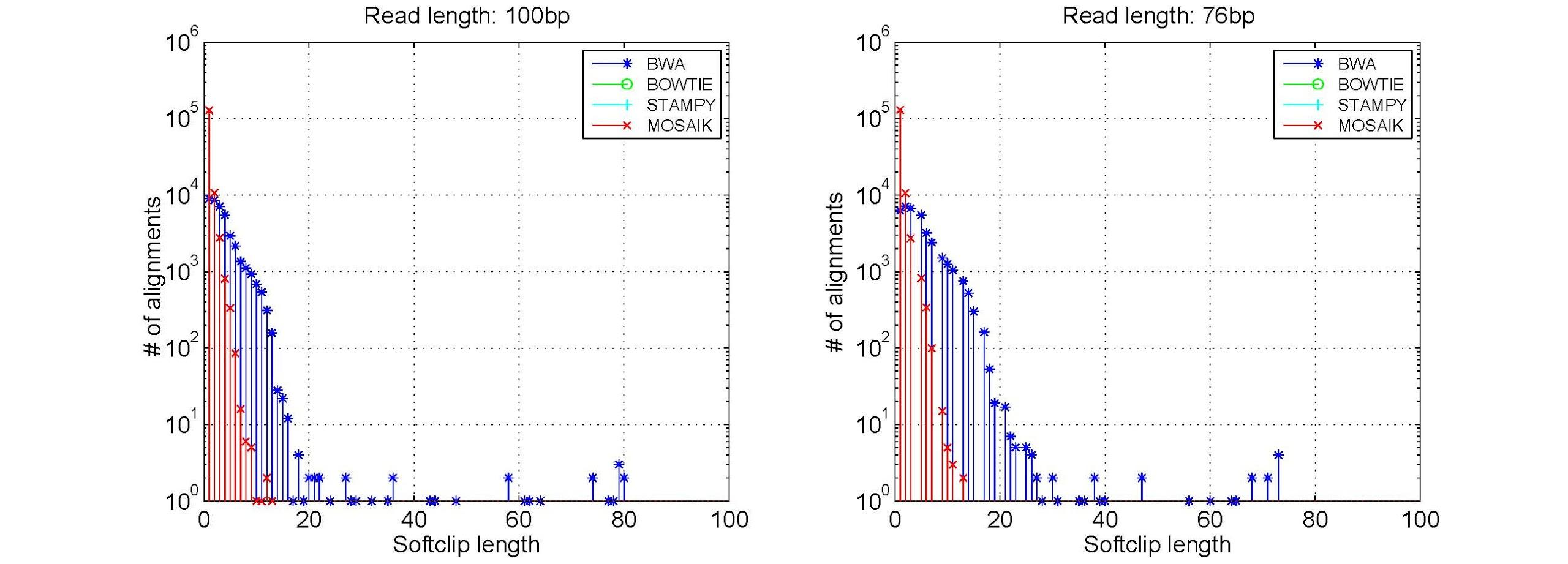

Supplement: Figure S1 — The distributions of alignments' softclips. (TIF) [file pone.0090581.s001.tif]

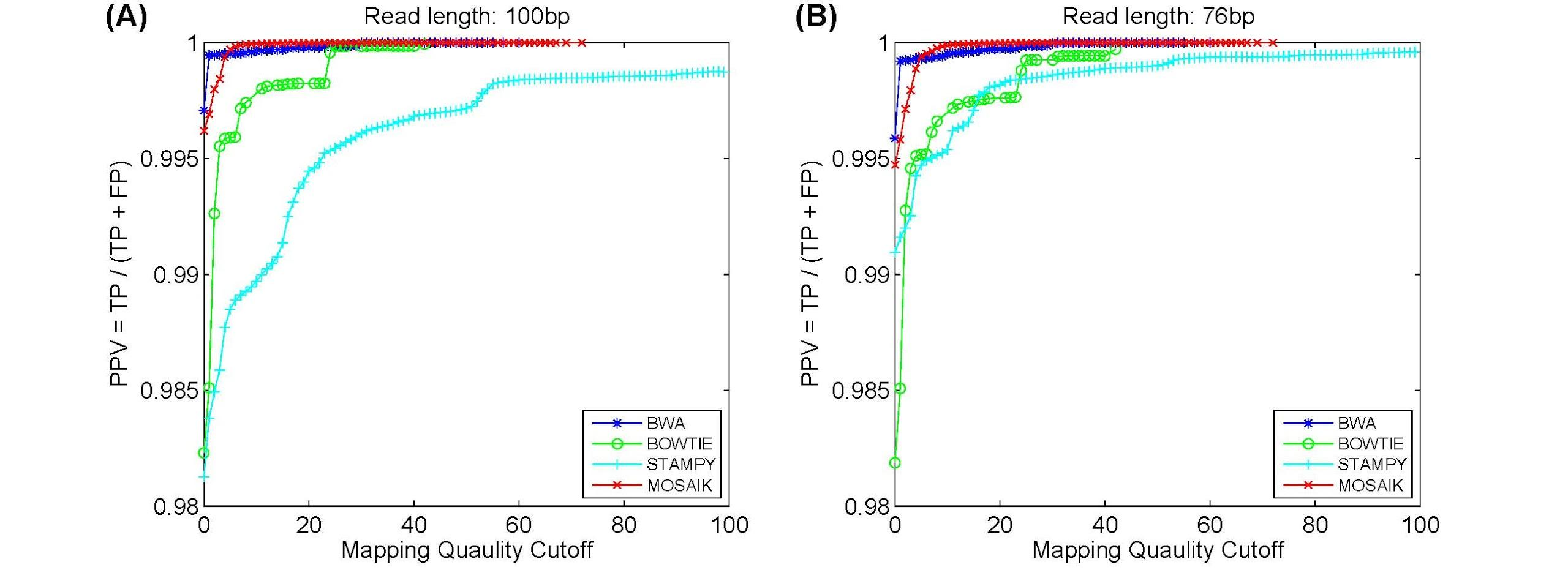

Supplement: Figure S2 — The complete information of Figure 1 . The positive predictive value of aligners (the number of correctly mapped reads divided by the total number of mapped reads) as a function of mapping quality threshold. Datasets in (A) 100 bp and (B) 76 bp read lengths. PPV, TP, and FP stand for positive predictive value, true positive, and false positive, respectively. (TIF) [file pone.0090581.s002.tif]

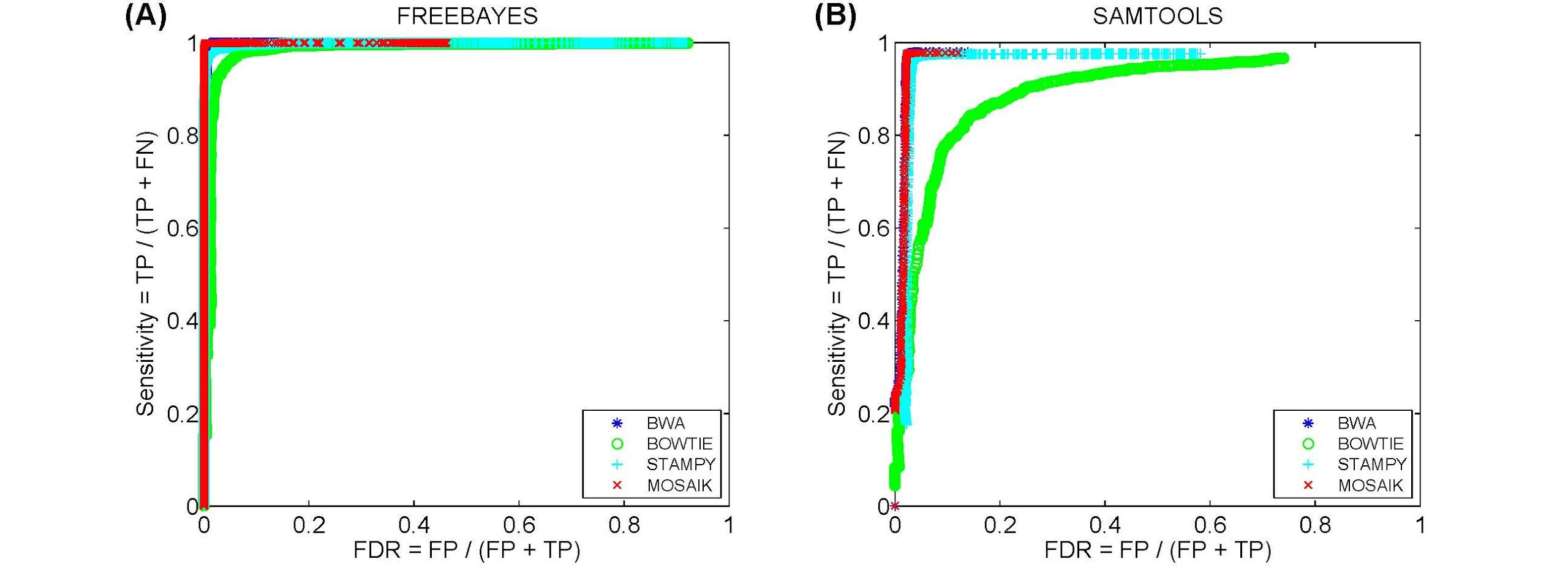

Supplement: Figure S3 — The complete information of Figure 5 . The receiver operating characteristic (ROC) curves of SNPs called by FREEBAYES and SAMTOOLS. The points on the curves are sorted by called qualities and the points closer to the upper-right corner have higher called qualities. The true positive (TP), false positive (FP), and false negative (FN) are calculated by intersecting SNPs called on each aligner's alignments and gold SNPs called on the simulated alignments. (TIF) [file pone.0090581.s003.tif]

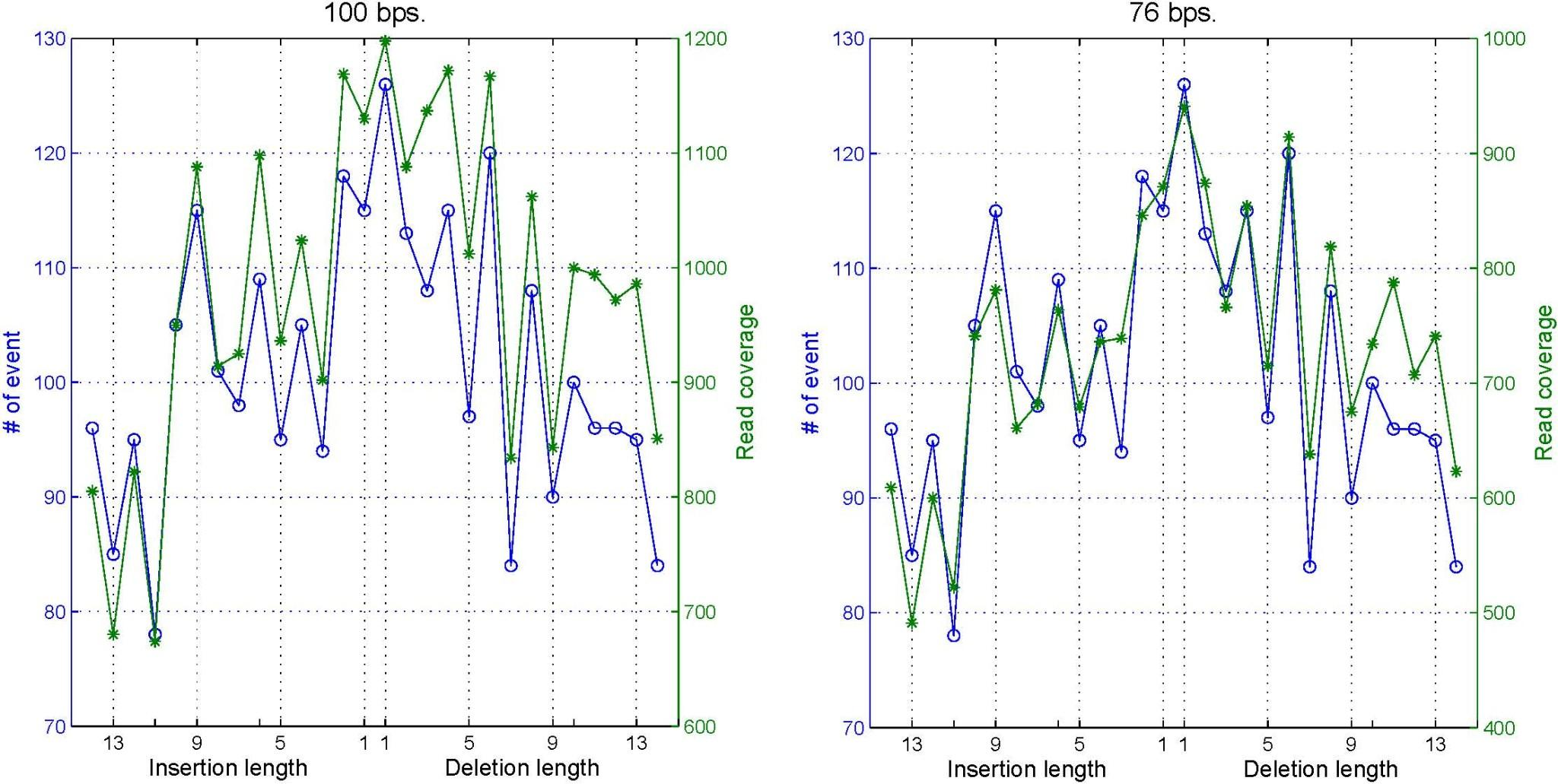

Supplement: Figure S4 — The short INDELs that are inserted for investigating the aligners' abilities for them, and the read coverage for each length INDEL. (TIF) [file pone.0090581.s004.tif]

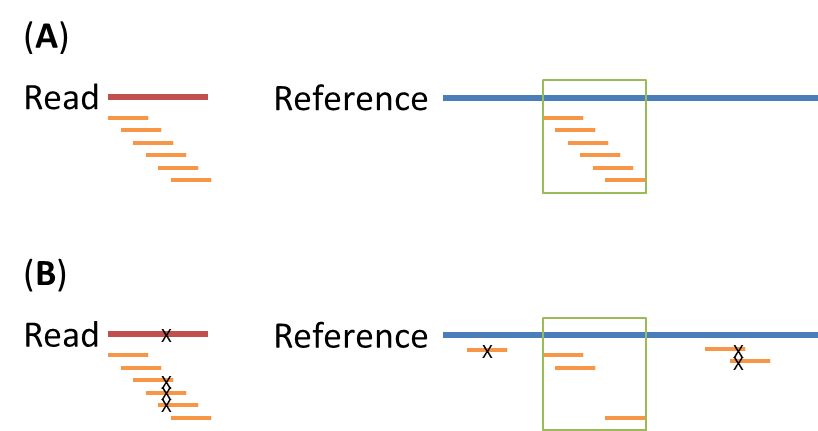

Supplement: Figure S5 — MOSAIK hash clustering. (A) The read uniquely aligns perfectly to the references, all hashes will succeed in finding the adjacent reference locations and the AVL tree will consolidate those hashes into one alignment candidate region. (B) However, if only one hash succeeds in finding the proper reference location because of sequencing errors, an alignment candidate region is still present in the AVL tree. (TIF) [file pone.0090581.s005.tif]

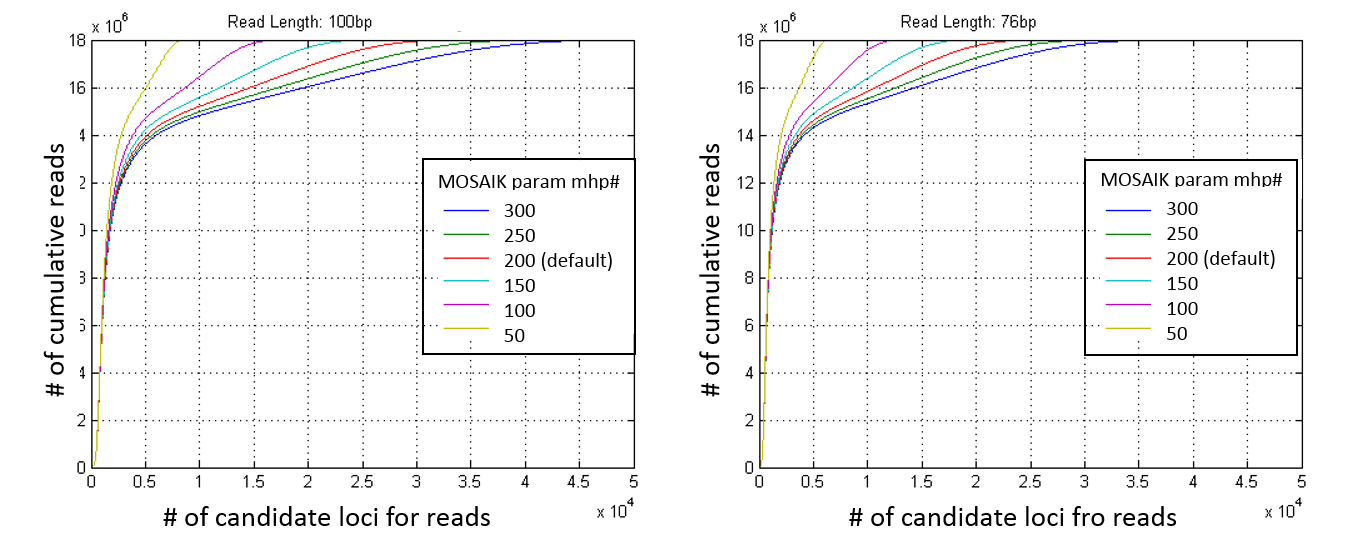

Supplement: Figure S6 — The distribution of candidate loci in the genome of reads. MOSAIK applies a Smith-Waterman algorithm to each candidate locus of a read to generate an alignment. Therefore, the number of candidate loci is equal to the number of executed the Smith-Waterman algorithm. The mhp of MOSAIK is the maximum number of investigated hash positions per 15-mer. (TIF) [file pone.0090581.s006.tif]
