# Supplementary material for: MOSAIK: A Hash-Based Algorithm for Accurate Next-Generation Sequencing Short-Read Mapping
Source: PLoS One. 2014 Mar 5;9(3):e90581. doi: 10.1371/journal.pone.0090581 (PMC3944147; doi:10.1371/journal.pone.0090581)
Supplement: Method S1 — The methods of (A) Retraining Mapping Quality Neural Network and (B) Detecting Specified Insertion Sequences. (PDF) [file pone.0090581.s007.pdf]

Supplemental Methods

(A) Retraining Mapping Quality Neural Network

We simulated 6 million paired-end reads from *E. coli* genome and aligned them by MOSAIK with "-zn" option that attach ZN optional tag for each alignment in the output bam-formatted file. A ZN tag consists of the best Smith-Waterman score, the next-best Smith-Waterman score, entropy of the read, length of the longest perfect match, the number of obtained alignments, and the number of obtained hashes. We further attached XC optional tag that shows the correct positions of reads. Then, we applied our training program to the files that have been attached ZN and XC. An example is given on https://github.com/wanpinglee/MOSAIK/blob/master/demo/RetrainMQ.sh.

(B) Detecting Specified Insertion Sequences

MOSAIK can be aware of alignments mapped to the specified insertion sequences. In a reference fasta-formatted file, users should attach insertion sequences with the same prefix. For example, we attached the mobile element insertions (MEIs) with the prefix "moblist_" after human genome references. Then, when aligning reads, we enable "-sref moblist" option to let MOSAIK move hashes located in the MEIs to the top. Consequently, aligning reads to MEIs is a priority by MOSAIK. The other option "-srefn <int>" can limit the number of hashes moved to the top. Once alignments are mapped to the specified insertions, MOSAIK will indicate that in ZA optional tags.
