# Supplementary material for: MOSAIK: A Hash-Based Algorithm for Accurate Next-Generation Sequencing Short-Read Mapping
Source: PLoS One. 2014 Mar 5;9(3):e90581. doi: 10.1371/journal.pone.0090581 (PMC3944147; doi:10.1371/journal.pone.0090581)
Supplement: Table S1 — The runtime of each mapper for aligning six million 100 bp reads. The version without ‘*’ are the exact version of each mapper for which we report performance comparisons. For up to date information, we also report speed for the current version (indicated by ‘*’) of each software. STAMPY is a single-threaded program and thus the runtime of using 4 cpus is not available. (PDF) [file pone.0090581.s008.pdf]

Supplemental Table

Table S1: The runtime of each mapper for aligning six million 100bp reads. The version without '*' are the exact version of each mapper for which we report performance comparisons. For up to date information, we also report speed for the current version (indicated by '*') of each software. STAMPY is a single-threaded program and thus the runtime of using 4 cpus is not available.

	**Run time; 4 cpus**	**Run time; 1 cpu**
BOWTIE (2.0-beta5)	15m 6.547s	42m 23.591s
BWA (0.5.9)	26m 21.311s	69m 52.714s
MOSAIK (2.1.78)	89m 48.887s	378m 15.979s
STAMPY (1.0.13)	Not available	404m 11.122s
*BOWTIE (2.1.0)	17m 38.875s	45m 28.829s
*BWA (0.7.5a)	17m 18.782s	59m 46.641s
*MOSAIK (2.2.3)	17m 11.22s	67m 23.724s
*STAMPY (1.0.22)	Not available	569m11.897s
